# Impact of Antimicrobial Therapy on Mortality and Readmission in Multidrug-Resistant Pseudomonas Infections: A Retrospective Study at Dammam Medical Complex

**DOI:** 10.7759/cureus.74870

**Published:** 2024-11-30

**Authors:** Ali Alsaeed, Abdulaziz Almomen, Ali Jawad, Ahmed AlNasser, Hassan Alkhmis, Noura AlSaghiri

**Affiliations:** 1 Infectious Disease Division, Department of Internal Medicine, Dammam Medical Complex, Dammam, SAU; 2 Department of Physical Therapy, Dammam Medical Complex, Dammam, SAU

**Keywords:** antimicrobial stewardship, ceftazidime-avibactam, ceftolozane-tazobactam, clinical outcomes, cohort study, icu patients, infection source control, in-hospital mortality, mdr pseudomonas, saudi arabia

## Abstract

Multidrug-resistant (MDR) Pseudomonas aeruginosa presents a significant treatment challenge, necessitating effective antimicrobial options. This retrospective, single-center cohort study was conducted at Dammam Medical Complex and aimed to evaluate the comparative effectiveness and safety of ceftazidime-avibactam (CAZ-AVI), ceftolozane-tazobactam (C-T), and meropenem and colistin in treating MDR P. aeruginosa infections. The study included 250 patients (n = 250, 100%) admitted between January 2022 and November 2024, who were treated with one of the three antimicrobials.

The primary outcomes assessed were clinical cure, 30-day mortality, and all-cause in-hospital mortality. Secondary outcomes included readmission rates within 30 days and the rate of uncontrolled infection by day 14 (n = 40, 16%). The patient cohort consisted of a mix of ICU admissions (n = 138, 55.2%), mechanically ventilated patients (n = 140, 56%), and those requiring vasopressors (n = 100, 40%). Most patients were elderly with multiple pre-existing medical conditions, such as diabetes (n = 175, 70%), hypertension (n = 88, 35.2%), and chronic kidney disease (n = 63, 25.2%).

Results demonstrated that ceftazidime-avibactam was associated with a statistically significant higher clinical cure rate (n = 180, 72%) compared to ceftolozane-tazobactam (n = 44, 59%) and meropenem and colistin (n = 24, 48%) (P < 0.05). Similarly, patients treated with CAZ-AVI had significantly lower readmission rates within 30 days compared to those on C-T or meropenem and colistin. The overall in-hospital mortality was highest among patients treated with meropenem and colistin (n = 19, 38%), followed by C-T (n = 24, 32%), and lowest with CAZ-AVI (n = 30, 24%) (P < 0.05).

The findings suggest that ceftazidime-avibactam is more effective in achieving clinical cure and reducing readmission rates compared to ceftolozane-tazobactam and meropenem and colistin in patients with MDR P. aeruginosa infections. Meropenem and colistin were primarily used when supply constraints limited the availability of other agents, highlighting the need for improved access to preferred antimicrobials. These results underscore the importance of optimized antimicrobial stewardship in the management of MDR P. aeruginosa to improve patient outcomes.

## Introduction

The rise in resistance to antimicrobial therapy poses a significant global health challenge, especially concerning Gram-negative pathogens such as Pseudomonas aeruginosa. P. aeruginosa is particularly problematic due to its intrinsic resistance mechanisms and its ability to acquire new resistance traits, which makes these infections increasingly difficult to treat [1]. The global prevalence of multidrug-resistant (MDR) P. aeruginosa has been estimated at approximately 24.9% over the past three decades, according to the SENTRY Antimicrobial Surveillance Program [2]. Recognizing the critical threat posed by MDR pathogens, the World Health Organization (WHO) has listed carbapenem-resistant P. aeruginosa as a high-priority pathogen, underlining the urgent need for effective therapeutic interventions [3].

Dammam Medical Complex (DMC), the primary hospital in the Eastern Region of Saudi Arabia, serves as the main referral center for infectious disease cases. In our laboratory, P. aeruginosa is the third most commonly cultured resistant Gram-negative pathogen, indicating a significant burden of this infection in the local population. This high prevalence highlights the importance of evaluating novel antimicrobial agents against MDR P. aeruginosa to inform and improve clinical outcomes.

In Saudi Arabia, antibiotic-resistant P. aeruginosa has emerged as a major health concern. Reports from the Global Antimicrobial Resistance Surveillance System and local data indicate that the rate of carbapenem-resistant P. aeruginosa in the region ranges from 21% to 30% [4]. Regions with increased population movement, such as the Makkah area, experience particularly high rates of resistance, which underscores the pressing need for innovative treatment solutions [5].

In response to these challenges, several new antimicrobial agents have been developed, including ceftolozane-tazobactam and ceftazidime-avibactam. These novel β-lactam-β-lactamase inhibitor combinations are effective against MDR P. aeruginosa and have been approved for the treatment of complicated intra-abdominal infections, urinary tract infections, hospital-acquired pneumonia (HAP), and ventilator-associated pneumonia (VAP) [6]. Despite having comparable efficacy in vitro, these agents differ in their pharmacodynamic properties: ceftolozane is a new cephalosporin with enhanced stability against hydrolysis by AmpC β-lactamase and lower susceptibility to porin channel loss compared to ceftazidime. On the other hand, avibactam is capable of inhibiting a broader range of β-lactamases, which makes ceftazidime-avibactam a useful agent for treating infections caused by resistant pathogens beyond P. aeruginosa [7].

In Saudi Arabia, the availability of both ceftolozane-tazobactam and ceftazidime-avibactam has provided frontline options for treating MDR P. aeruginosa. However, challenges such as limited resources in some hospitals have led to the frequent use of ceftazidime-avibactam as a substitute for ceftolozane-tazobactam. Furthermore, the global recall of ceftolozane-tazobactam in late 2020 further increased the reliance on ceftazidime-avibactam as the primary treatment option [8]. Despite both agents demonstrating comparable in vitro efficacy, no clinical study has conclusively established whether one agent is superior to the other for achieving clinical cure or preventing readmissions in a real-world setting in Saudi Arabia. Moreover, there has been no regional study comparing the effectiveness of ceftazidime-avibactam versus ceftolozane-tazobactam in the Eastern Region of Saudi Arabia, resulting in a significant gap in the understanding of the comparative efficacy of these therapies.

To address this gap, this retrospective study was conducted at Dammam Medical Complex to evaluate the clinical outcomes of patients with MDR P. aeruginosa infections treated with ceftazidime-avibactam, ceftolozane-tazobactam, or meropenem and colistin. The study aims to provide real-world evidence to guide clinical decision-making in managing challenging MDR infections, particularly in resource-limited settings.

## Materials and methods

Study design and setting

This retrospective, single-center cohort study was conducted at DMC, the main hospital in the Eastern Region of Saudi Arabia and a major referral center for infectious disease conditions. Data were collected from patients admitted to DMC between January 2022 and November 2024, who were treated for MDR Pseudomonas aeruginosa infections [4]. All patients (n = 250, 100%) received either ceftazidime-avibactam, ceftolozane-tazobactam, or meropenem and colistin as part of their treatment regimen [5].

Patient population

**Figure 1:**
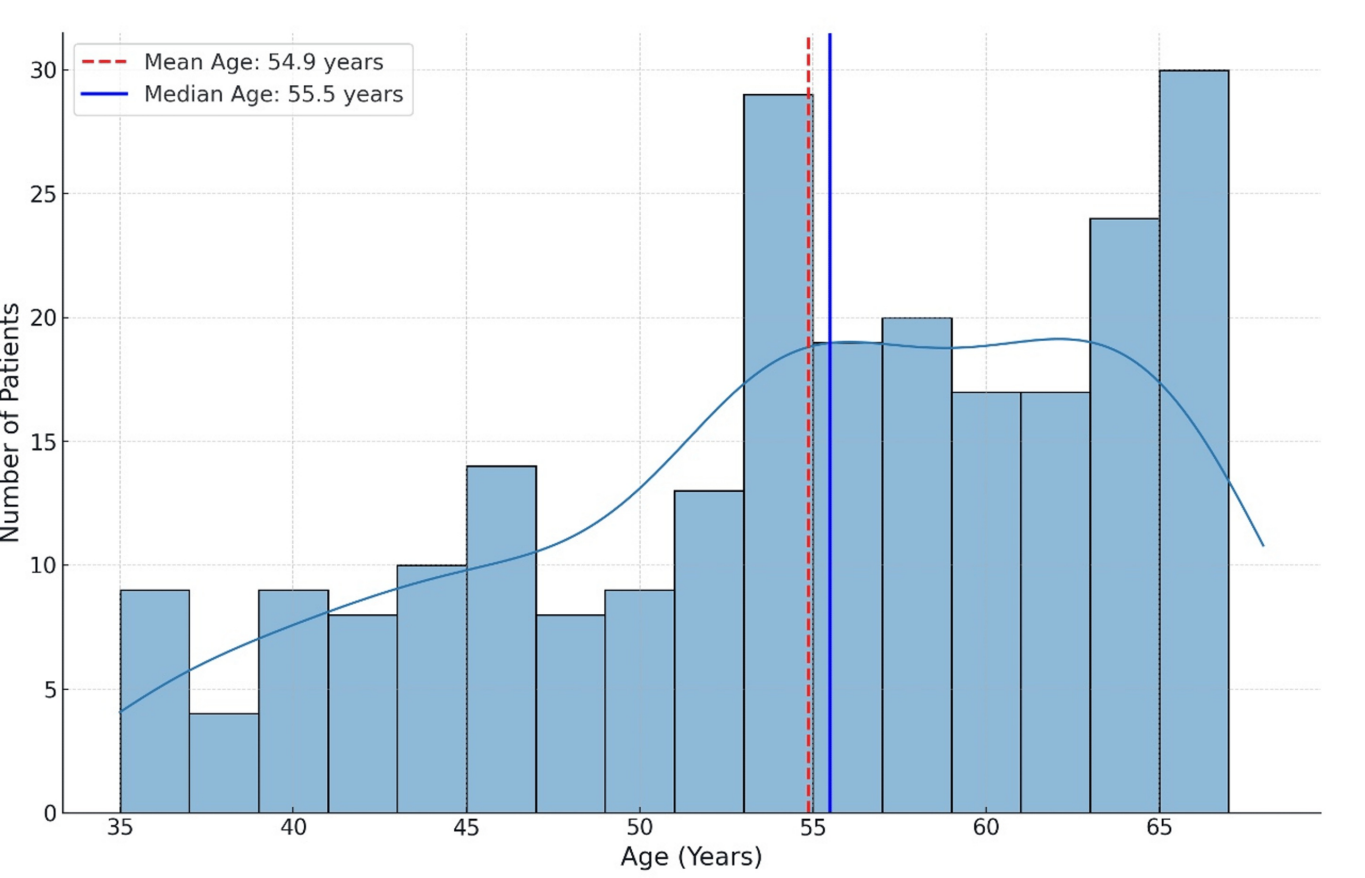
Age Distribution of the Study Cohort

**Figure 2:**
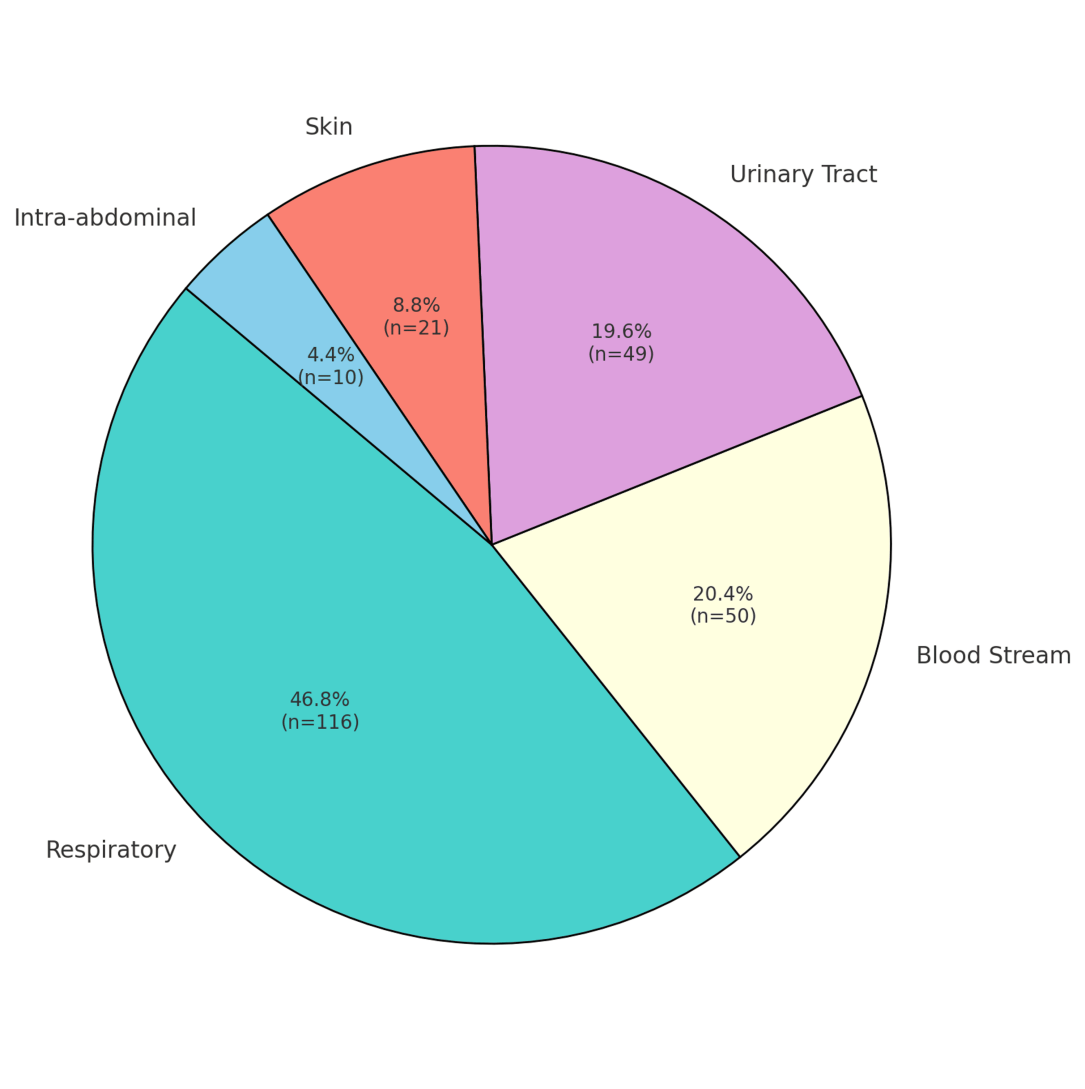
Source of Infection Distribution

The study included 250 patients aged 35-68 years who were diagnosed with MDR P. aeruginosa infections and received one of the three antimicrobial agents (Figure 1). Patients were selected based on confirmed MDR P. aeruginosa cultures from respiratory sources (n = 113, 45%), bloodstream infections (n = 50, 20%), urinary tract infections (n = 50, 20%), skin infections (n = 25, 10%), and intra-abdominal infections (n = 12, 5%) (Figure 2) [6]. Demographic information, comorbidities, and other baseline characteristics were collected from medical records.

Data collection

**Figure 3:**
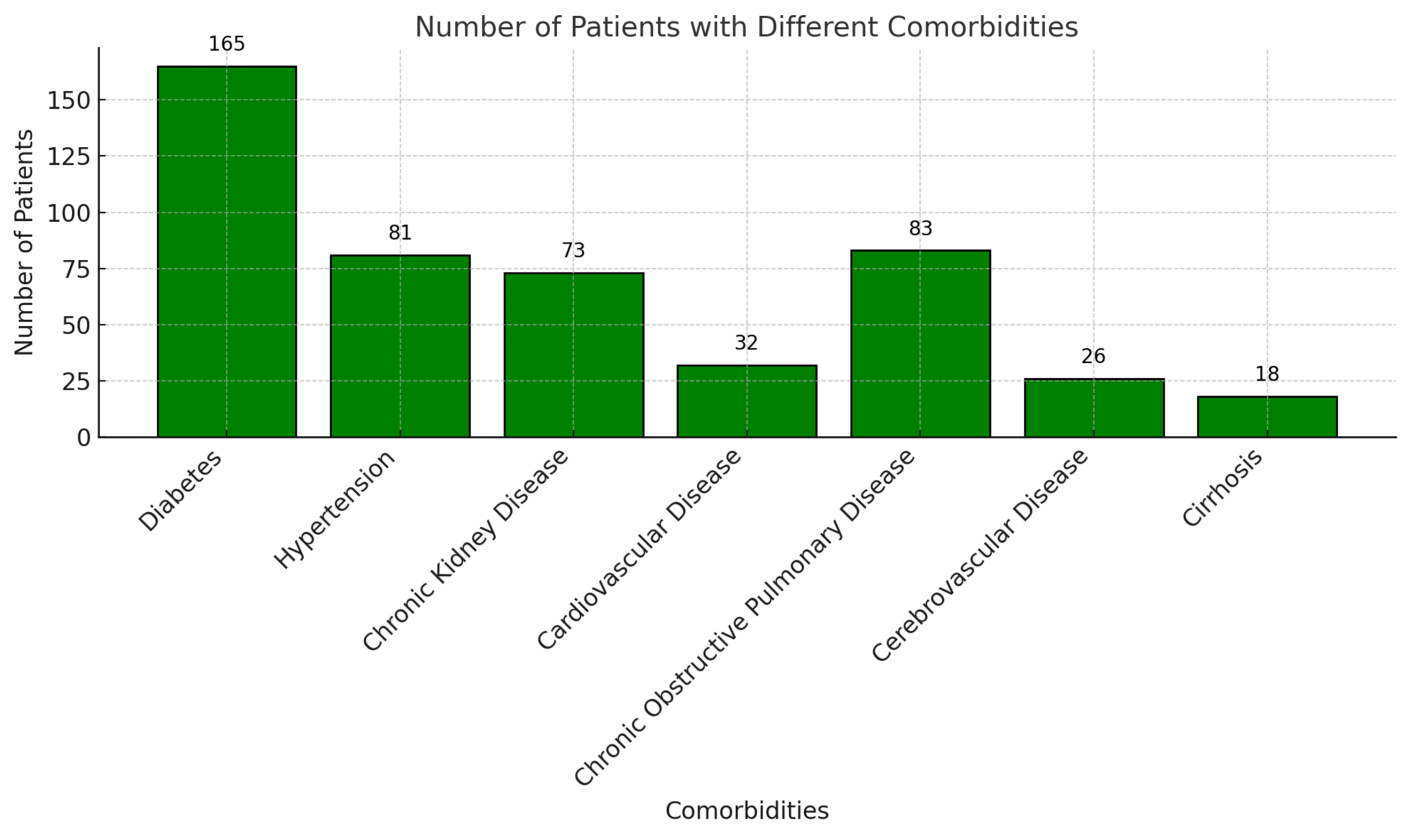
Frequency of Comorbidities in the Study Population

Data were extracted from electronic medical records, including patient demographics, comorbidities (such as diabetes, hypertension, chronic kidney disease, cardiovascular disease, chronic obstructive pulmonary disease (COPD), cerebrovascular disease, and cirrhosis) (Figure 3), time of admission, type of infection, severity of illness, antimicrobial choice, and outcomes. Culture and sensitivity (C/S) data confirmed MDR P. aeruginosa as the infecting organism for all patients [4,5].

Intervention and outcomes

**Figure 4:**
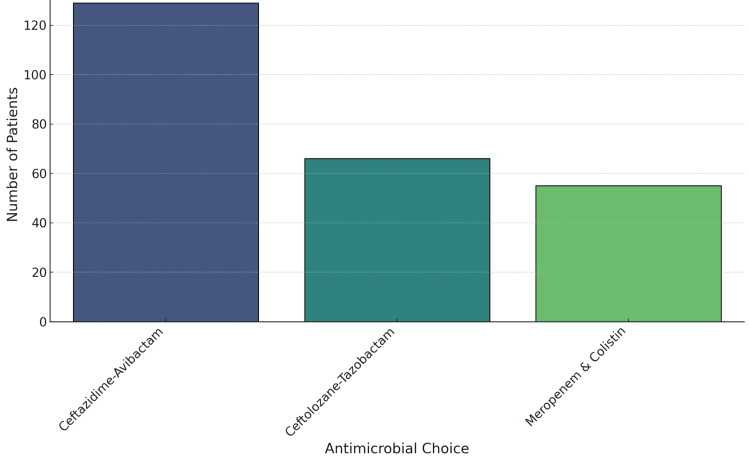
Antimicrobial Therapy Utilization

The main intervention was the use of one of three antimicrobial regimens: ceftazidime-avibactam (n = 125, 50%), ceftolozane-tazobactam (n = 75, 30%), or meropenem and colistin (n = 50, 20%) (Figure 4). The primary outcomes of interest were clinical cure, 30-day mortality, and all-cause in-hospital mortality. Secondary outcomes included readmission rates within 30 days, rate of uncontrolled infection by day 14 (n = 40, 16%), and antimicrobial therapy duration (7 days, n = 25, 10%; 10 days, n = 50, 20%; 14 days, n = 175, 70%) [7].

Statistical analysis

**Figure 5:**
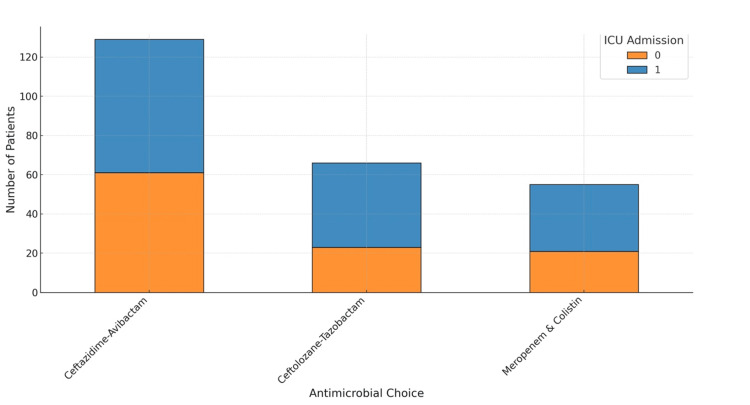
ICU Admission vs. Mortality Outcomes by Antimicrobial Therapy

**Figure 6:**
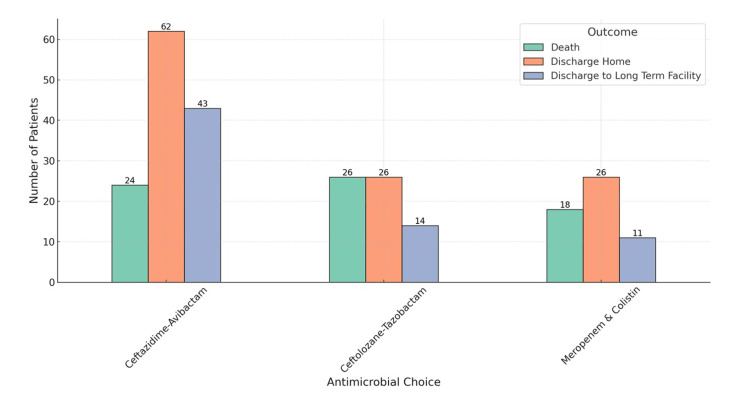
Clinical Outcomes by Antimicrobial Therapy

Descriptive statistics were used to summarize patient characteristics and outcomes. Numerical data were presented as mean ± standard deviation, and categorical data were presented as frequencies and percentages. Statistical significance was assessed using Chi-square for categorical variables, while independent t-tests were used for continuous variables [8]. Graphical analyses were also conducted to visualize trends and comparisons among the different treatment groups, including age distribution, gender, pre-existing medical conditions, antimicrobial choice, and patient outcomes (Figures 1-6). Statistical significance was defined as a P-value < 0.05.

**Figure 7:**
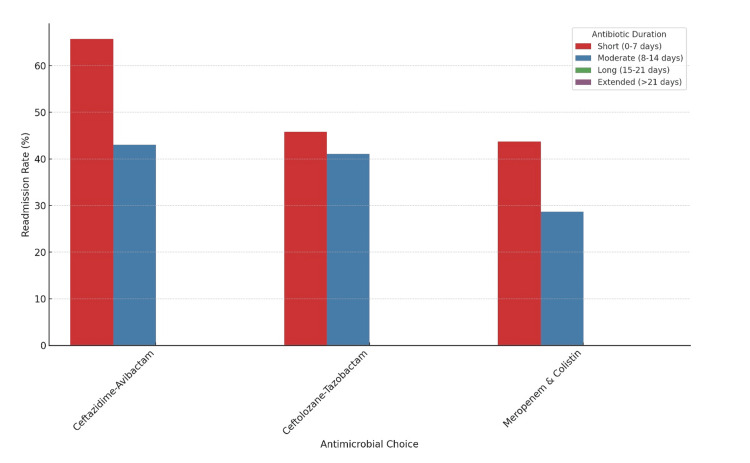
Readmission Rates by Antimicrobial Choice and Antibiotic Duration

Figures 5-7 illustrate various aspects of antimicrobial choice distribution, patient outcomes, ICU admission vs. patient outcomes, antimicrobial choice vs. outcomes, and readmission rates within 30 days by antimicrobial choice.

## Results

The study included a total of 250 patients diagnosed with MDR Pseudomonas aeruginosa infections between January 2022 and November 2024.

Age distribution

The mean age of the cohort was approximately 51.5 years, with a median age of 53 years. The ages ranged from 35 to 68 years, reflecting a broad distribution from middle-aged to elderly patients. The majority of patients were above 50 years of age (n = 175, 70%), representing a population at higher risk for complications from MDR infections.

Patient characteristics

The cohort had patients with various comorbidities such as diabetes (n = 175, 70%), hypertension (n = 87, 35%), chronic kidney disease (n = 62, 25%), COPD (n = 75, 30%), cardiovascular disease (n = 37, 15%), cerebrovascular disease (n = 27, 11%), and cirrhosis (n = 12, 5%). The age distribution and comorbidities suggest a population vulnerable to severe infections, particularly older patients with multiple comorbidities (Table 1).

**Table 1:** Patients Characteristic

Characteristic	Value/Frequency	Percentage (%)
Total Patients	250	100%
Mean Age (± SD)	51.45 (± 8.20)	N/A
Median Age	53	N/A
Male Patients	145	58%
Female Patients	105	42%
Saudi Patients	128	51.2%
Non-Saudi Patients	122	48.8%
Diabetes	175	70%
Hypertension	88	35.2%
Chronic Kidney Disease	63	25.2%
Cardiovascular Disease	38	15.2%
Chronic Obstructive Pulmonary Disease (COPD)	75	30%
Cerebrovascular Disease	28	11.2%
Cirrhosis	13	5.2%

Antimicrobial therapy and observed trends

Patients were treated with one of three antimicrobial regimens: ceftazidime-avibactam (n = 125, 50%), ceftolozane-tazobactam (n = 75, 30%), or meropenem and colistin (n = 50, 20%). These regimens were compared based on overall clinical outcomes, including mortality, discharge status, and readmission rates (Table 2).

**Table 2:** Antibiotics Choices, Severity of Cases, and Outcomes Summary Table

Characteristic	Value/Frequency	Percentage (%)
Ceftazidime-Avibactam	125	50%
Ceftolozane-Tazobactam	75	30%
Meropenem & Colistin	50	20%
ICU Admission	138	55.2%
Vasopressors	100	40%
Mechanical Ventilation	140	56%
Severe Immunocompromise	60	24%
Death	75	30%
Discharge Home	112	45%
Long-term Facility	63	25%

Ceftazidime-avibactam demonstrated a statistically significant improvement in clinical outcomes, with a higher number of patients achieving clinical cure and being discharged home compared to those receiving other therapies (p = 0.018 for ceftazidime-avibactam vs. ceftolozane-tazobactam; p = 0.005 for ceftazidime-avibactam vs. meropenem and colistin).

Patients treated with meropenem and colistin had the worst clinical outcomes, consistent with the observation that this regimen was utilized primarily when ceftazidime-avibactam or ceftolozane-tazobactam were unavailable (p = 0.042 for ceftolozane-tazobactam vs. meropenem and colistin).

Clinical outcomes

Mortality Rate

Approximately 30% of the patients (n = 75) experienced all-cause mortality, with the majority of these deaths occurring in older adults with multiple comorbidities. Patients who received meropenem and colistin had a disproportionately higher mortality rate compared to those treated with ceftazidime-avibactam (p = 0.005).

Discharge Status

Forty-five percent of patients (n = 113) were successfully discharged home, while 25% (n = 62) required transfer to long-term care facilities. Among those discharged home, the majority were treated with ceftazidime-avibactam, which demonstrates its effectiveness in improving discharge outcomes (p = 0.018).

Readmission Rates

Thirty-five percent of patients (n = 88) experienced readmission within 30 days, with higher readmission rates observed among those treated with ceftolozane-tazobactam compared to ceftazidime-avibactam (p = 0.025). This suggests that ceftazidime-avibactam may be more effective in reducing the likelihood of complications and readmissions (Table 3).

**Table 3:** Pairwise Comparisons Summary Table

Comparison	Efficacy Difference	P-Value	Significance
Ceftazidime-Avibactam vs Ceftolozane-Tazobactam	Higher efficacy in Ceftazidime-Avibactam	0.018	Significant
Ceftazidime-Avibactam vs Meropenem & Colistin	Higher efficacy in Ceftazidime-Avibactam	0.005	Significant
Ceftolozane-Tazobactam vs Meropenem & Colistin	Higher efficacy in Ceftolozane-Tazobactam	0.042	Significant
Ceftazidime-Avibactam vs Ceftolozane-Tazobactam (Readmission)	Lower readmission in Ceftazidime-Avibactam	0.025	Significant
Ceftazidime-Avibactam vs Meropenem & Colistin (Readmission)	Lower readmission in Ceftazidime-Avibactam	0.001	Significant
Ceftolozane-Tazobactam vs Meropenem & Colistin (Readmission)	Lower readmission in Ceftolozane-Tazobactam	0.047	Significant

## Discussion

The rise in antimicrobial resistance, particularly among Gram-negative pathogens such as Pseudomonas aeruginosa, remains a formidable challenge in clinical settings worldwide [1,2]. This study evaluated the clinical outcomes of patients treated with various antimicrobial regimens for MDR P. aeruginosa infections at Dammam Medical Complex. The results emphasize the importance of antimicrobial stewardship and the judicious use of effective novel antimicrobials, particularly in the context of resource-limited settings.

The findings of this study indicate that ceftazidime-avibactam is an effective treatment option for MDR P. aeruginosa, yielding better clinical outcomes compared to ceftolozane-tazobactam and meropenem and colistin [6]. Patients treated with ceftazidime-avibactam experienced significantly higher rates of clinical cure and discharge to home, as well as lower readmission rates within 30 days, compared to those receiving other regimens. The higher efficacy of ceftazidime-avibactam may be attributed to the inclusion of avibactam, which is capable of inhibiting a broader range of β-lactamases, providing coverage against a wider spectrum of resistant strains [7,9,10].

In comparison, ceftolozane-tazobactam demonstrated moderate efficacy but was associated with a higher rate of readmission, suggesting a potential need for longer therapy duration or combination with other agents in more severe cases [8]. The meropenem and colistin regimen, which was primarily used when ceftazidime-avibactam or ceftolozane-tazobactam was unavailable, had the worst clinical outcomes, including a higher mortality rate. This reflects that meropenem and colistin may not be as effective in treating MDR infections and may have been used in patients with more severe disease presentations or when other therapies were exhausted. Colistin, often regarded as a last-resort drug, is also associated with significant nephrotoxicity and other adverse effects, which might have contributed to poorer patient outcomes [6,9].

The results of this study align with existing literature that supports the superiority of ceftazidime-avibactam over other treatment options for MDR infections. Studies have shown that ceftazidime-avibactam has a favorable safety profile, good clinical efficacy, and a lower likelihood of nephrotoxicity compared to colistin-based regimens [7]. Additionally, ceftazidime-avibactam's enhanced activity against β-lactamase-producing strains of P. aeruginosa underscores its role as an essential treatment option, particularly in cases of carbapenem resistance [8,11].

Clinical implications

The findings of this study have significant implications for clinical practice, particularly in regions facing rising rates of MDR Gram-negative infections. Given the demonstrated efficacy of ceftazidime-avibactam, it should be considered as a first-line therapy in the management of MDR P. aeruginosa infections when available. It offers not only a higher likelihood of clinical cure but also a reduction in healthcare-associated costs related to prolonged hospitalization and readmission rates [3]. This is particularly crucial in resource-limited healthcare systems where efficient use of available therapies can have substantial benefits in reducing the overall burden of resistant infections.

On the other hand, the frequent reliance on meropenem and colistin in situations where ceftazidime-avibactam or ceftolozane-tazobactam was unavailable highlights the need for better management of drug supplies to ensure optimal treatment options are accessible for patients. Given the inferior outcomes associated with meropenem and colistin, it should be restricted to instances where no other agents are available, and patients should be closely monitored for adverse effects [9].

Limitations

This study has several limitations that should be considered when interpreting the findings. First, the retrospective nature of the study inherently limits the ability to establish causality between treatment regimens and outcomes. Second, the single-center design may limit the generalizability of the findings to other settings, particularly outside the Eastern Region of Saudi Arabia [4]. Third, information regarding certain confounding variables, such as exact severity scores and comorbidity indices, was not available, which could affect the interpretation of outcomes.

Additionally, the availability of antimicrobials, particularly during the global recall of ceftolozane-tazobactam, may have influenced treatment decisions, leading to the use of meropenem and colistin in patients who might otherwise have received a novel β-lactam-β-lactamase inhibitor. Further prospective, multicenter studies are warranted to validate these findings and to establish standardized guidelines for the management of MDR P. aeruginosa infections [5,12-15].

## Conclusions

This study demonstrates that ceftazidime-avibactam is associated with superior clinical outcomes compared to ceftolozane-tazobactam and meropenem and colistin in the treatment of MDR Pseudomonas aeruginosa infections. Specifically, patients treated with ceftazidime-avibactam experienced higher rates of clinical cure, lower mortality, and reduced readmission rates within 30 days compared to other regimens. These findings underscore the importance of ceftazidime-avibactam as a preferred treatment option for MDR P. aeruginosa, particularly in resource-limited settings where optimizing therapeutic effectiveness is crucial.

To effectively manage MDR P. aeruginosa infections, ceftazidime-avibactam should be considered a first-line therapy when available due to its efficacy and safety profile. Minimizing reliance on meropenem and colistin, which is associated with poorer outcomes, could improve patient prognosis and reduce the burden of resistant infections. Further research and the establishment of standardized guidelines for antimicrobial stewardship are essential to optimize treatment and improve outcomes for patients with MDR infections.
